# The Phosphatic Index: Its Value to Us in the Healing Art

**Published:** 1913-10

**Authors:** Edward M. Dooley

**Affiliations:** Attending: Surgeon Sister’s Mercy, and Emergency Hospitals; Surgeon Buffalo Creek and Nickel Plate railroads


### The Phosphatic Index: Its Value to Us In the Healing Art

EDWARD M. DOOLEY, M. D Attending Surgeon Sister's Mercy, and Emergency Hospitals; Surgeon Buffalo Creek and Nickel Plate railroads.

IN the New York Medical Record, May, 1908, Dr. J. Henry Dowd gave to the profession his original article, "The Phosphatic Index or Pulse of the Nervous System."

Up to this time practically nothing had appeared that might act as an aid in ascertaining the condition of a system that controls and directs every movement of the human body, sick or well.

In presenting the subject it must be admitted as yet scientific points may be lacking, but the therapeutic results have more than born out the immense value of the procedure to be described.

Empiricism, as this may be considered by some, is most aptly combatted by a few paragraphs taken from an article by Sir Dyce Duckwith, British Medical Journal, May, 1911: "Let us have a right conception of empirical. No less an authority than Sir William Hamilton has defined it in these terms, 'Experience founded on observation alone.' As applied to a legitimate practice of medicine in these days it cannot be said that empiricism is experiment or experience without science, for the phenomena of disease are now carefully studied by many men well trained in scientific methods, and the results of treatment are no less rigidly noted as a scientist would note them.

We take note of dogmatic opinions issued from the laboratories in regard to the action of drugs, and articles of diet which in some cases completely contradict the experience of clinical experts.

For example, we find a condemnation of alcohol as a food and remedy at the hands of physiologists who have no clinical practice or experience of the treatment of morbid conditions in their fellow creatures. The physician replies that he is sadly aware of the ill-effects of alcohol when improperly used by man, and the toxic and deadly effects in large doses when used on small animals, but in clinical practice he finds it exceedingly useful for his patients in certain conditions.

In a like manner we are told that strychnine has no action on the heart, and that aconite is of no avail in reducing the frequency of the pulse, but in clinical practice good results come somehow in the use of these drugs."

With the above opinion from men of the type mentioned, we cannot discard results that have been obtained merely on the ground that it is empirical, for we must not forget that in many conditions empiricism has often anticipated science in medicine. It is known, generally speaking, that urea represents the metabolic process of the muscular system; phosphorus in its various combinations—known as phosphates—represents the metabolism of the nervous system. Phosphorus is one, and, no doubt, the most important constituent of the nervous system, in fact a great scientist has said, "When all the phosphorus is taken, the human race will cease to exist." Phosphorus, together with its associates, lecithin and nuclein occur in the urine as phosphates, being end products of the above mentioned. Phosphates appear as acid sodium phosphate and acid calcium and magnesium phosphate. Phosphoric acid is partly derived from alkaline and earthly phosphates and partly as a decomposition of lecithin and nuclein, these substances being also taken from the food. In speaking of the phosphates found in the urine, the alkaline variety, those precipitated by an alkaline solution are the ones to be considered, the phosphates occuring in freshly passed urine, or phosphatic crystals have no consideration in the subjects under discussion, they may be ignored.

Professor John B. Shaw in his work on Nervous Diseases says, referring to neurasthenia, a condition that can cover practically all conditions, "This inherited unstable state of the nervous system depends evidently upon some defect in the power of the neurons to assimilate and store up nutrition and force in sufficient quantities and with sufficient rapidity to carry on fully and easily the work of life." We must, therefore, assume that nerve cells prepare for their work or outlay of energy by food that nourish them and in common with other structures of the body are capable of accumulating a reserve which can be held in store for times of emergency, times when through illness sufficient food is not taken, or from other causes when consumption is not equal to the demand.

Have we not a practically analogous condition with other organs; there are two eyes, lungs, kidneys, etc., one is only absolutely necessary, the other is the reserve.

The all important substances necessary in the nourishment and upbuilding of the nervous system are phosphorus, lecithin and nuclein, these are taken from our food, and providing digestion and assimilation are normal, sufficient quantity is taken, not only for the daily calls for energy, but an excess which is stored.

Although phosphates vary at different parts of the day and to a certain extent by diet, it will be found that irritation of the nervous system, especially hypersensibility or irritability of the nerve cells plays the most important part in increasing them. If this is allowed to continue to any degree, it will in time cause a depletion of the reserve ending in variable systemic prostration due to nerve cell starvation; this is commonly called neurasthenia.

J. C. Clemesha, in the New York Medical Journal, in his article, "The Dowd Phosphatic Index: Its Relation to Diseases of the Eye," most aptly sums up this condition as follows:

.1 Normal output of phosphates with normal size and shape of crystals showing the metabolism of the nervous system in health and normal conditions.

2. Excessive output of phosphates, shown by high phosphatic index, an increased metabolism of the nervous system due to hypersensibility or irritability of nerve cells; the crystals are generally small in size.

3. Decreased output of phosphates shown by a low index, a condition due to a lack of nuclein and lecithin reserve.

4. We may have the normal amount of phosphates, but the crystals are light in weight though normal in size, showing an ill regulated nervous balance wheel. Amorphous crystals are found at times, and show a degeneration of nerve tissue.

The phosphatic elimination or phosphatic index was determined by Dr. Dowd after an extensive series of urinary examinations from people in as normal a state of health as possible, and free from all nerve irritation. The index was made from the second urine passed in the morning, and if correct readings are to be obtained with the phosphatometer this point must be strictly adhered to. The following points will be of great value in arriving at the true condition.

Anything that markedly increases the urine, such as chilling the body, diuretics or liquor, must be taken into consideration and another sample used if necessary.

The same may be said of a decreased quantity.

When in an apparently healthy individual the index is very high, enquiry should be made as to a possible shock, or irritation of the nervous system shortly before the sample was passed —it may be only transitory.

Where the crystals all appear amorphous it is well to enquire as to the patient's previous twenty-four hour conduct, a late supper with liquor, tobacco, etc., in excess will so change the crystals that all may appear amorphous, yet there may not be any degenerative changes going on.

The modus operandi is very simple, and is as follows:

Fill phosphatometer to "U," add solution (this accompanies the instrument) to "S," and set aside for ten minutes. At once a precipitate should form varying in degrees of density according to the amount of phosphates present. It is important that the reading should not be made until the expiration of ten minutes.

A plus index SOLID above "NP" indicates an irritation of the nerve cells, nervous metabolism is increased and will continue so unless sedatives are given and the cause removed.

A minus index, one that will not sink, sinks part way and is light and fluffy or goes below "NP," indicates nerve cell starvation, and no matter what may be the case under consideration, but little or no improvement will be forthcoming until the nerve cells are supplied with proper nourishment.

The consideration of nerve food is a very important one, inasmuch as when phosphorus is indicated, it must be given in its elimentary form and not as a compound in pills or tablets. Of late much has been written on this subject and all agree (J. A. M. M. and others) that phosphorus is not obtainable from hypo, or glycerophosphates, phosphoric acid mixtures from tablets and pills.

Besides the Phosphatometer, we, as physicians, owe much to Dr. Dowd as the chemist who gives us a preparation which contains free phosphorus as is proven by smell, taste, fumes, its luminosity in the dark.

There seems to be no one place for the Phosphatometer and the phosphatic index judging from the reported cases of Drs. Bennet, Clemesha, Decot and other oculists; Diehl in skin; Chauncey P. Smith in surgery; Walsh, Talbot of Niagara Falls and Kelly of Grand Rapids in general medicine, and Haines of Pennsylvania in gynæcology. In fact, as Dr. Smith says, "The Phosphatic index should be a routine in all medical examinations, as it gives more information than practically any other scientific test we have at hand; Phosphorus, when given in its free state, gives immediate and permanent results."

On account of the great number of cases reported already, many of which have been treated for some time without results and in which there was almost immediate improvement after treatment as directed by the phosphatometer, I will add but one case, and in doing so must state, this patient had been treated for some time without any result whatever. Mrs. L.—There was no organic trouble, the condition was purely functional, but to a degree that living was almost a burden. Symptoms varied at times, neuritis was most marked, involving different parts of the body in succession, the bladder suffering most and the condition accompanied by frequency of urination and great pain. There was also a marked dyspepsia accompanied by gas accumulation. Space will not permit me to give a detailed report. She remarked, "I scarcely see a well day." On taking her index it was found to be 90 per cent minus, a condition of nerve cell starvation being plainly shown. The Dowd Comp. Phos. mixture was ordered in 30 drop doses three times a day, about 30 to 40 minutes after food. The improvement in this case was marvelous, inside of 48 hours she reported improvement, in two weeks she was entirely free from pain, failing strength had returned, appetite increased, also weight, all dyspeptic symptoms had vanished. This patient was well two years afterward.

1192 Main St., Buffalo, N. Y.

